# Protective effects of a food-grade recombinant *Lactobacillus plantarum* with surface displayed AMA1 and EtMIC2 proteins of *Eimeria tenella* in broiler chickens

**DOI:** 10.1186/s12934-020-1297-4

**Published:** 2020-02-11

**Authors:** Qiong Liu, Yanlong Jiang, Wentao Yang, Yongshi Liu, Chunwei Shi, Jing Liu, Xing Gao, Haibin Huang, Tianming Niu, Guilian Yang, Chunfeng Wang

**Affiliations:** 1 College of Animal Science and Technology, Jilin Provincial Engineering Research Center of Animal Probiotics, Key Laboratory of Animal Production and Product Quality Safety of Ministry of Education, Jilin Agricultural University, 2888 Xincheng Street, Changchun, 130118 China; 2 College of Food Engineering, Jilin Engineering Normal University, 3050 KaiXuan Road, Changchun, 130052 Jilin China

**Keywords:** *Lactobacillus plantarum*, Food-grade, Surface displayed expression, *Eimeria tenella*

## Abstract

**Background:**

Avian coccidiosis posts a severe threat to poultry production. In addition to commercial attenuated vaccines, other strategies to combat coccidiosis are urgently needed. *Lactobacillus plantarum* has been frequently used for expression of foreign proteins as an oral vaccine delivery system using traditional erythromycin resistance gene (*erm*). However, antibiotic selection markers were often used during protein expression and they pose a risk of transferring antibiotic resistance genes to the environment, and significantly restricting the application in field production. Therefore, a food-grade recombinant *L. plantarum* vaccine candidate would dramatically improve its application potential in the poultry industry.

**Results:**

In this study, we firstly replaced the erythromycin resistance gene (*erm*) of the pLp_1261Inv-derived expression vector with a non-antibiotic, *asd*-*alr* fusion gene, yielding a series of non-antibiotic and reliable, food grade expression vectors. In addition, we designed a dual-expression vector that displayed two foreign proteins on the surface of *L. plantarum* using the anchoring sequences from either a truncated poly-γ-glutamic acid synthetase A (pgsA′) from *Bacillus subtilis* or the *L. acidophilus* surface layer protein (*SlpA*). EGFP and mCherry were used as marker proteins to evaluate the surface displayed properties of recombinant *L. plantarum* strains and were inspected by western blot, flow cytometry and fluorescence microscopy. To further determine its application as oral vaccine candidate, the AMA1 and EtMIC2 genes of *E. tenella* were anchored on the surface of *L. plantarum* strain. After oral immunization in chickens, the recombinant *L. plantarum* strain was able to induce antigen specific humoral, mucosal, and T cell-mediated immune responses, providing efficient protection against coccidiosis challenge.

**Conclusions:**

The novel constructed food grade recombinant *L. plantarum* strain with double surface displayed antigens provides a potential efficient oral vaccine candidate for coccidiosis.
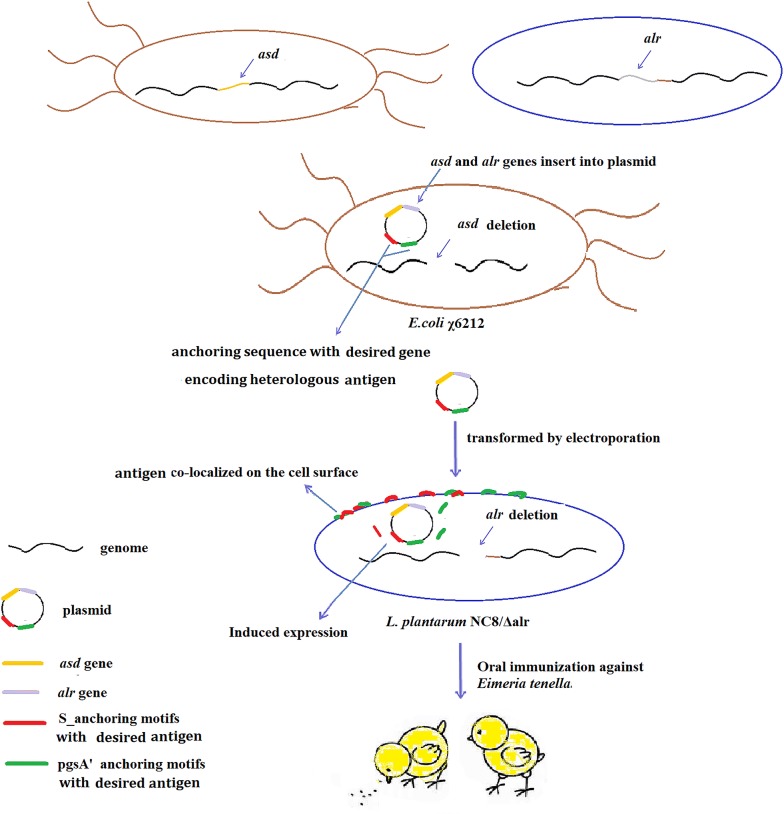

## Background

Chicken coccidiosis is an intestinal protozoan disease caused by the parasite *Eimeria*, causing serious economic losses to poultry industry worldwide [1]. The most effective means of controlling the disease relies on prophylactic anti-coccidial drugs. However, the increase of parasites resistant to anti-coccidial drugs and the public pressure to limit the use of chemicals in animals [2] have prompted the development of cost-effective vaccines. Although some commercial vaccines are available, such as live virulent, attenuated [3] or live-tolerant vaccines [4], some disadvantages have been noticed including the poor immunological protection, affecting weight gain and virulence reversion [5]. To overcome these problems, several novel vaccine candidates have been studied recently. In particular, bacterial vectors originating from but not limited to *Bacillus subtilis* [6], *Mycobacterium bovis* BCG [7], *Salmonella enteritidis* [8] and *Lactococcus lactis* [9] have drawn more and more attention to be used as vectors for oral vaccines.

Among these vectors, lactic acid bacteria (LAB) have long been used in the fermentation industry as probiotics with the ability to enhance the functionality of the immune system. LAB has also great potential to serve as a vehicle for oral vaccine delivery, by genetically expressing desired foreign proteins [10]. There are three different ways to present foreign proteins (or antigens), which include intracellular, cellular surface and secreted protein production. Usually it is considered that the cell surface anchored antigen and secreted antigen could induce much stronger immune responses compared with the intracellular production of desired antigen due to the higher degree of exposure of the antigen to the host. Our lab has recently used the anchoring sequence from poly-γ-glutamic acid synthetase A (pgsA), a transmembrane protein from *Bacillus subtilis* [11–13] in order to construct recombinant *L. plantarum* strains capable in expressing foreign antigens. The resulting strains were efficient in the production of protective antibody responses and partial protection against either influenza virus [14], transmissible gastroenteritis virus [15, 16] or porcine epidemic diarrhea virus [17]. Notably, the traditional recombinant *L. plantarum* vaccines still relies on antibiotic resistance markers such as erythromycin, ampicillin or kanamycin. However, more and more studies have shown that the antibiotic resistance genes may not be safe for food or oral vaccine applications due to its possibility to affect environment [18]. To overcome these disadvantages, several non-antibiotic selection markers such as *thy*A [19], *thr* [20], and *alr* [21] have been developed. Another commonly used non-antibiotic selection marker is the *asd* gene (EC1.2.1.11), which has been used in *E. coli* and *Salmonella* [22].

Apical membrane antigen 1 (AMA1) is a member of the highly conserved parasite surface proteins among apicomplexan protozoan, such as *Toxoplasma gondii* [23], *Plasmodium* and *Coccidia* [24]. The AMA1 is found on the sporozoite surface of *E. tenella*. Its expression level at sporozoite stage is higher than that of other stages. The AMA1 protein is a type I transmembrane protein, which plays an important role in sporozoite invasion [25]. A previous report showed that DNA vaccines encoding EbAMA1 of *E. brunetti* could increase the level of serum specific IgG and cytokines concentration [26]. AMA1 of *E. tenella* sporozoite is effective at stimulating partial protection against a homologous challenge when expressed as a recombinant protein by *Lactococcus lactis* [27].

In addition, a number of microfilament proteins such as EtMIC2, which is expressed throughout the life cycle of coccidia and located on the membrane of sporozoites, can be used as potential vaccine candidate antigens against *E. tenella* infection by either eukaryotic or prokaryotic vectors [28–30]. Protective efficacy analysis indicated EtMIC2 conveys only partial protection [31]. To improve the immunogenicity and protective efficacy of EtMIC2 and AMA1, the two antigens were co-expressed on the surface of *L. plantarum*. As a vaccine delivery vector, *L. plantarum* has the function of enhancing immunity. We speculate that AMA1 and EtMIC2 co-expressed by *L. plantarum* could stimulate immune responses and improve immune protection against *E. tenella* infection.

In this study, we firstly designed a non-antibiotic dependent expression system for use in *L. plantarum* strains using the *asd* and *alr* selective markers. Then a double anchoring vector consisting of pgsA′ and S-layer protein anchoring sequences was constructed and the AMA1 and EtMIC2 proteins of *Eimeria tenella* (*E. tenella*) were anchored on the surface of recombinant *L. plantarum* strain Lp-12 at the same time. The results of in vivo studies suggested that the novel double-antigen anchoring construction delivered via *L. plantarum* significantly enhanced the immune response in chicken, which protected against *E. tenella* challenge.

## Results

### Construction of non-antibiotic selective plasmids

**Fig. 1:**
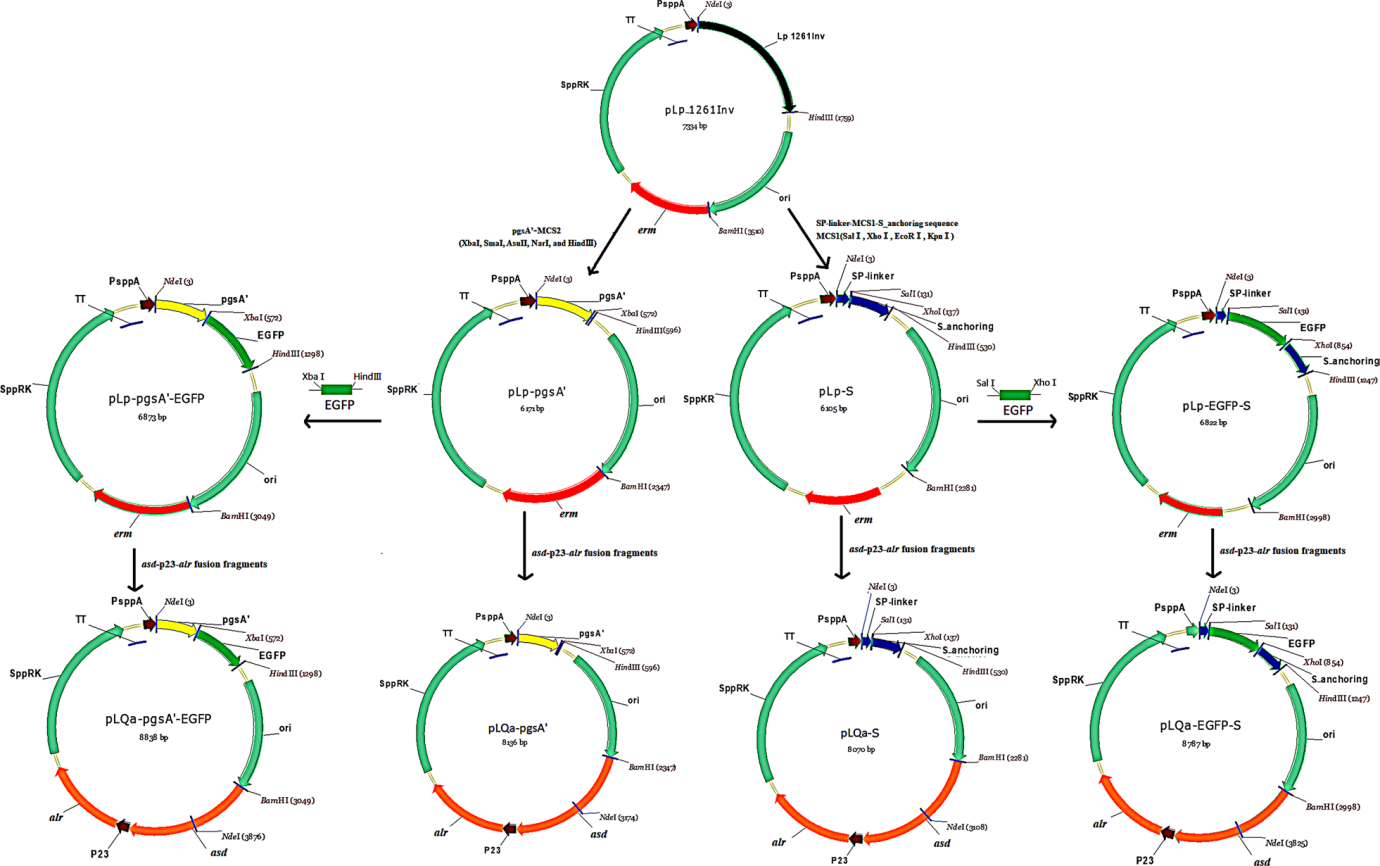
Construction process of *erm*-marked and *asd*-*alr*-marked plasmids for the expression of single-anchored EGFP

In this study, we sought to create a single expression vector that could be used to co-express two foreign proteins using a dual-anchoring sequence and non-antibiotic selection markers. To achieve this goal, we constructed a series of vectors, including vectors with a food-grade marker (*asd*-*alr*) containing various anchoring sequences (see Fig. 1). The Lp1261inv gene in parental plasmid pLp_1261Inv was replaced with either a pgsA′ or S anchor encoding sequence, yielding pLp-pgsA′ or pLp-S, respectively. Then EGFP gene was inserted into the above plasmids, resulting in two EGFP anchoring plasmid pLp-pgsA′-EGFP and pLp-EGFP-S. On the other hand, the *erm* resistant gene was replaced with an *asd*-*alr* expression cassette, which could synthesize Asd and Alr at the same time, yielding the non-antibiotic selective plasmids pLQa-pgsA′, pLQa-S, pLQa-pgsA′-EGFP and pLQa-EGFP-S.

**Fig. 2:**
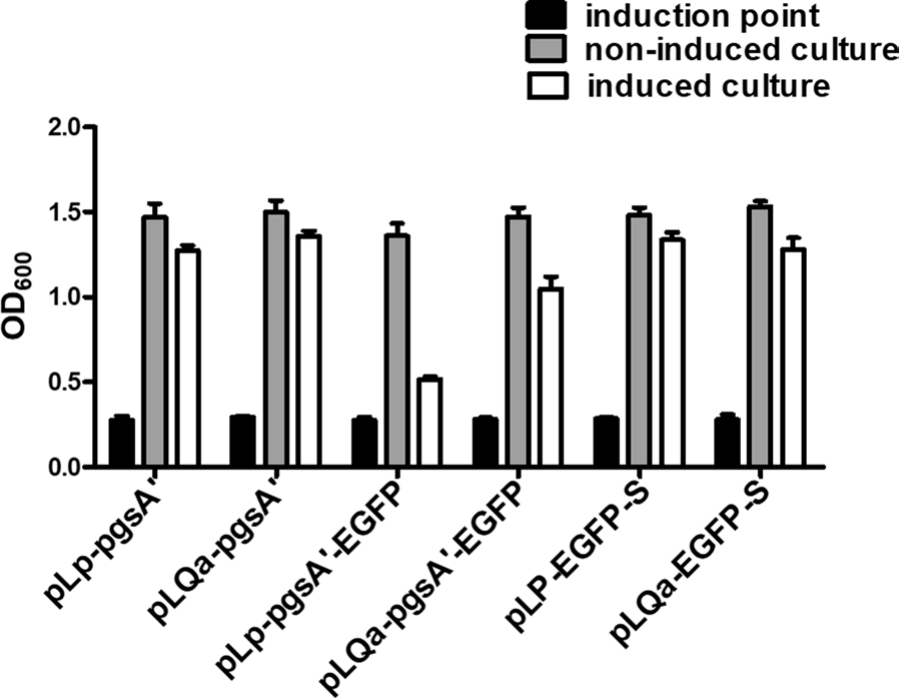
Growth characteristics of EGFP-expressing *L. plantarum* strains. Bacterial cells were pre-cultured to OD_600_ ≈ 0.3. MRS was divided into two equal parts, one in non-induced culture (gray bars) and another in induced culture with 25 ng/mL SppIp (white bars). OD_600_ values were measured 4 h post induction. pLP-pgsA′, pLQa-pgsA′ pLp-S and pLQa-S are the empty vectors without the target proteins, and we only used the pLP-pgsA′ or pLQa-pgsA′ as empty vector control

To determine whether the expression of foreign proteins affects the growth of recombinant strains, the constructed plasmids described above were transformed into either *L. plantarum* NC8 or its *alr* deletion mutant strain *L. plantarum* NC8/Δalr. After induction by the addition of SppIp, significantly decreased growth rates were observed for strains containing pLp-pgsA′-EGFP or pLQa-pgsA′-EGFP, whereas the growth rates of strains harboring pLp-EGFP-S or pLQa-EGFP-S were not obviously affected (Fig. 2). The reason of the decreased growth for strains containing pLp-pgsA′-EGFP or pLQa-pgsA′-EGFP is not clear. We speculate that the pgsA’ anchoring sequence plays an important role that affects growth rate, because the growth rates of the strains containing the pgsA’ sequence were lower than those containing the S_anchoring sequence. PgsA’ is a transmembrane-anchoring sequence. Interestingly, a previous study also showed that the Lp_1568 transmembrane anchoring sequence significantly affected the growth of *L. plantarum* [32]. Our results suggest that the S layer anchoring approach may be a better choice for surface displayed expression in *L. plantarum* strains.

### Synthesis of EGFP on surface of recombinant strains

**Fig. 3:**
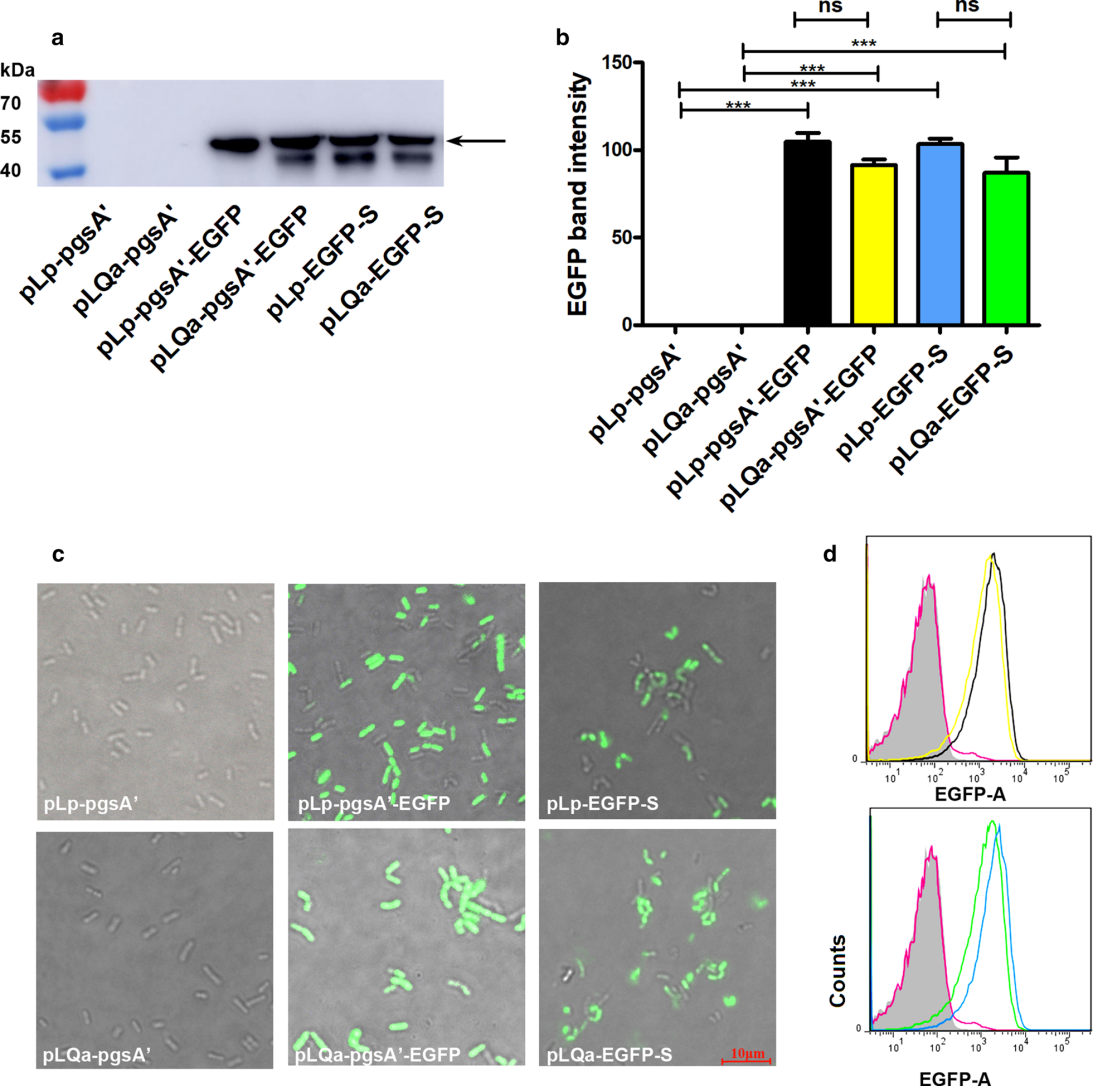
**a** Western blot analysis of EGFP. Same amount of total protein was loaded for each strain. Two strains containing pLP-pgsA′ (Lane 1) and pLQa-pgsA′ (Lane 2) were used as negative controls. The arrow indicates the bands of the EGFP fusion proteins. **b** Quantitative analysis of EGFP fusion proteins from Western blots. **c** Fluorescence microscopy analysis of single-anchoring expression of EGFP in *L. plantarum* containing *asd*-*alr*-marked or *erm*-marked plasmids (×100 objective). **d** Representative images of flow cytometry analysis of recombinants containing the following plasmids at 4 h post induction: pLp-pgsA′-EGFP (black), pLQa-pgsA′-EGFP (yellow), pLP-EGFP-S (light blue), pLQa-EGFP-S (green). pLP-pgsA′ (pink) and pLQa-pgsA′ (gray) were used as negative controls

The production and display of EGFP on the surface of *L. plantarum* strains was determined by Western blot, fluorescence microscopy and flow cytometric analysis. As expected, a 46 kDa band (26.3 kDa for EGFP plus the anchoring sequence) was observed in all constructs harboring the EGFP encoding gene (Fig. 3a). A second band with lower molecular weight was observed in some samples (lanes 4–6, but not 3 in Fig. 3a). It is not clear what this band is, but one possibility is that this is a putative protein product, as previously reported [32–35]. The strength of the respective bands on the Western blot showed no significant difference between the *erm*-marked and *asd*-*alr*-marked plasmids (Fig. 3b). The production of EGFP was further confirmed by fluorescence microscopy (Fig. 3c) and flow cytometry (Fig. 3d). All results demonstrated that the synthesis of EGFP was not dramatically affected by the replacement of the *erm* with an *asd*-*alr* cassette, indicating the possibility to use non-antibiotic selective markers for further application.

### Stability of non-antibiotic selective strains

Next, we tested the stability of both *erm* and *asd*-*alr*-selective plasmids. The results showed that the percentage of transformed NC8 strains still harboring the plasmids pLp-pgsA′-EGFP and pLp-EGFP-S were 83% and 86% after 100 generations in selective medium (MRS with erythromycin), respectively. On the other hand, all plasmids were lost in the absence of antibiotics after 100 generations (Table 1). Notably, the percentages of remaining plasmids pLQa-pgsA′-EGFP and pLQa-EGFP-S in NC8/Δ*alr* strains achieved about 66% and 69% at the presence of d-ala after 100 generations, whereas 100% plasmids were found in NC8/Δ*alr* strains when d-ala was absent in culture medium, suggesting that the *alr* selective marker may benefit the stability of recombinant *L. plantarum* strains.

**Table 1:** Stability of *erm*-marked and *asd*-*alr*-marked plasmids

Plasmid/medium	50 generations	100 generations
	Percentage containing plasmid	Percentage containing plasmid
pLP-pgsA′-EGFP/MRS	46	0
pLP-EGFP-S/MRS	48	0
pLQa-pgsA′-EGFP/MRS	100	100
pLQa-EGFP-S/MRS	100	100
pLp-pgsA′-EGFP/MRS + erythromycin	91	83
pLp-EGFP-S/MRS + erythromycin	93	86
pLQa-pgsA′-EGFP/MRS + d-ala	83	66
pLQa-EGFP-S/MRS + d-ala	85	69

^a^*L. plantarum* NC8 containing pLp-pgsA′-EGFP and pLP-EGFP-S, respectively, were cultivated in MRS solid media with or without 5 μg/mL erythromycin

^b^*L. plantarum* NC8/Δ*alr* containing pLQa-pgsA′-EGFP and pLQa-EGFP-S, respectively, were cultivated in MRS solid media with or without 200 μg/mL d-ala. These strains were sub-cultivated at 30 °C without agitation and passaged every 12 h. The percentage containing plasmid was calculated as the number of single colonies on selected plates

### Expression of EGFP and mCherry in a single plasmid with co-anchoring sequences

**Fig. 4:**
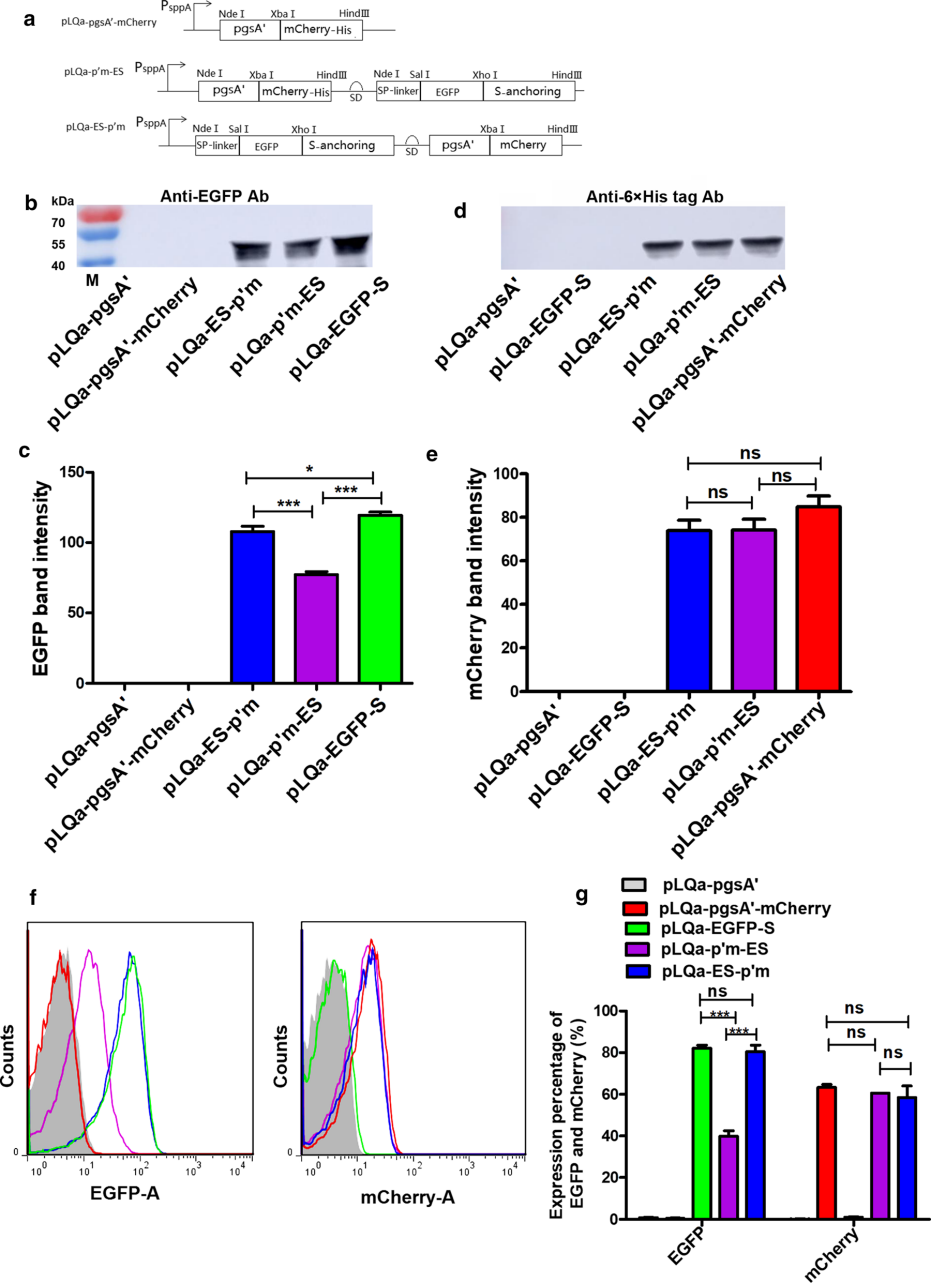
**a** The expression cassette for single- or dual-anchored proteins. The promoter PsppA mediated single-anchoring of the mCherryAT in pLQa-pgsA′-mCherry. Each of the co-anchoring expression plasmids (pLQa-p′m-ES and pLQa-ES-p′m) contains the inducible promoter PsppA, followed by two connected single-anchoring cassettes, which were separated by the SD sequence (AGGAAACAGACC). The difference between pLQa-p′m-ES and pLQa-ES-p′m is the splicing order of each anchoring cassette. **b** Western blot of EGFP using mouse anti-EGFP monoclonal antibody. **c** Quantitative analysis of western blot shown in **b**. **d** Western blot of mCherry using mouse anti-6 × His tag monoclonal antibody. **e** Quantitative analysis of western blot shown in **d**. **f** Representative images of flow cytometric analysis of *L. plantarum* NC8/*Δalr* strains containing the plasmids designed for single- or dual-anchoring of EGFP or mCherry. The strains are denoted by different colors in the flow cytometry histograms: pLQa-EGFP-S (green), pLQa-pgsA’-mCherry (red), pLQa-p’m-E (purple), pLQa-ES-p’m (blue). EGFP-A represents the EGFP-expressing strains; mCherry-A represents the mCherry-expressing strains. The *L. plantarum* NC8/*Δalr* harboring the empty pLQa-pgsA’ plasmid was used as a negative control (gray). (**h**) Statistical analysis of the flow cytometry results obtained by one-way ANOVA (*NS*: no significant difference, P > 0.05, ***P < 0.001)

The cloning cassettes of single- or dual-anchored EGFP and mCherry proteins in plasmids pLQa-pgsA′-mCherry, pLQa-p′m-ES and pLQa-ES-p′m are shown in Fig. 4a. A Shine-Dalgarno (SD) sequence (AGGAAACAGACC), which helps recruit ribosomes to the messenger RNA to initiate protein synthesis, was used for the expression of the secondary protein gene, either EGFP in pLQa-p′m-ES or mCherry in pLQa-ES-p′m.

Expression of EGFP and mCherry was confirmed with anti-EGFP and anti-6 × His Tag antibodies. Western blot revealed the presence of a 45.4 kDa band for EGFP (26.3 kDa of EGFP plus the anchoring sequence) (Fig. 4b). The quantitative analysis indicated that the expression level of EGFP was significantly higher in the single-anchor plasmid (pLQa-EGFPS) (P < 0.05) compared with the dual-anchor plasmid (pLQa-p′m-ES) (Fig. 4c). The presence of a 47.6 kDa band corresponds to mCherry (26.6 kDa for mCherry) plus the His-tag and anchoring sequence (Fig. 4d). The identity of this band was further confirmed by western blot, where no significant differences among single or double expression plasmids could be observed (Fig. 4e). These findings were also confirmed by flow cytometry analysis (Fig. 4f, g). All these results indicated that the synthesis of the desired protein in double expression plasmid was mainly dependent on the type and order of anchors rather the target protein.

**Fig. 5:**
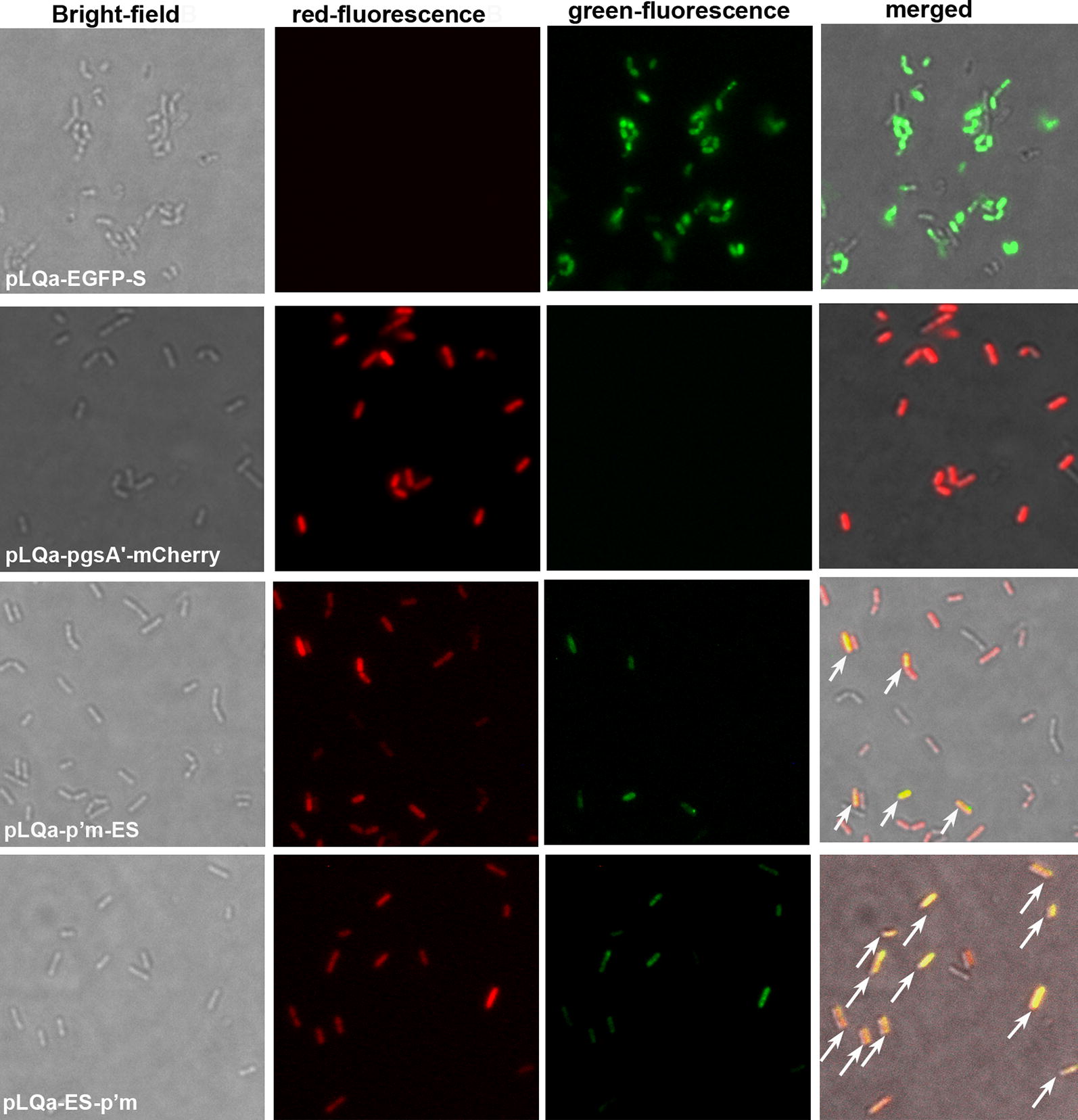
Fluorescence microscopy showing co-anchored EGFP and mCherry encoded by a single plasmid. *L. plantarum* NC8/Δ*alr* harboring plasmids pLQa-EGFP-S or pLQa-pgsA′-mCherry designed for single-anchoring of mCherry or EGFP were used as controls (×100 objective). White arrows indicate co-localization of EGFP and mCherry in strains harboring pLQa-p′m-ES and pLQa-ES-p′m, respectively

Co-expression of EGFP and mCherry was further confirmed by fluorescence microscopy (Fig. 5). No green-fluorescence or red-fluorescence was observed in *L. plantarum* NC8/Δ*alr* strains harboring the pLQa-EGFP-S or pLQa-pgsA′-mCherry for single-anchoring either EGFP or mCherry. On the other hand, both green-fluorescence and red-fluorescence were detected in NC8/Δ*alr* strains harboring plasmids pLQa-p′m-ES and pLQa-ES-p′m for co-anchoring EGFP and mCherry. When the green and red fluorescence were merged together, the recombinants were yellow for *L. plantarum* NC8/Δ*alr* harboring pLQa-p′m-ES or pLQa-ES-p′m (Fig. 5), indicating that they were colocalized.

### Co-anchoring of AMA1 and EtMIC2 on surface of *L. plantarum* strains

**Fig. 6:**
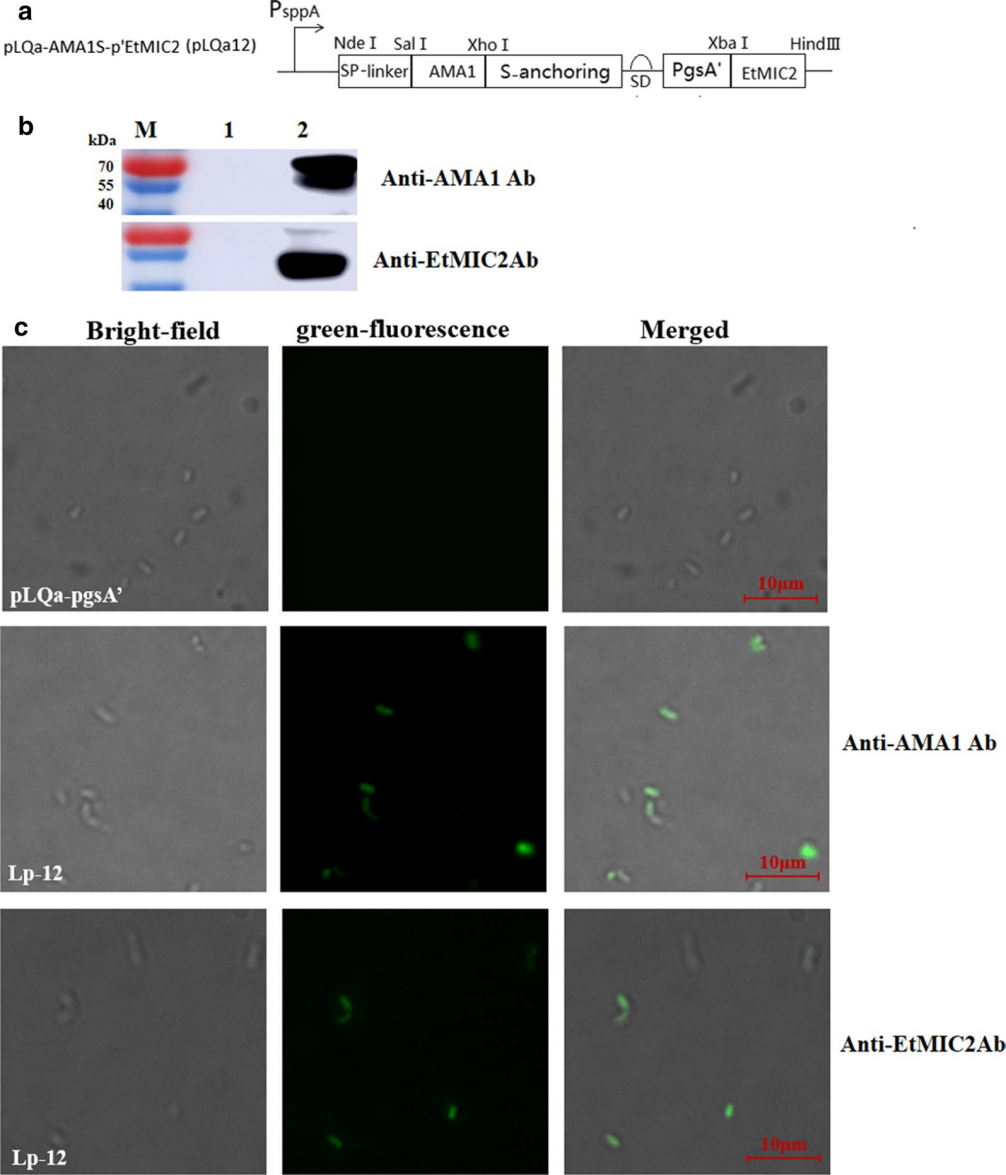
**a** The expression cassette for co-anchoring of AMA1 and EtMIC2 in the plasmid pLQa-AMA1S-p′EtMIC2. **b** Synthesis of AMA1 and EtMIC2 proteins in Lp-12 was assessed by Western blotting. Lane M: Marker; Lane 1: Lp/pLQa-pgsA′; Lane 2: Lp-12. **c** Immunofluorescence analysis to detect co-anchored AMA1 and EtMIC2 on the surface of *L. plantarum*

According to the results shown in Fig. 5, we selected the construct “protein1-S anchoring-SD-pgsA′-protein2” for further study. Two antigens of *E. tenella* (AMA1 and EtMIC2) were inserted into the plasmid pLQa-ES-p′m to obtain pLQa-AMA1S-p′EtMIC2, named pLQa12 (Fig. 6a). The *L. plantarum* NC8/Δalr containing the pLQa12 was referred as Lp-12. Protein expression of AMA1 and EtMIC2 on the surface of Lp-12 surface was determined by western blotting and immunofluorescence using anti-AMA1 or anti-EtMIC2 antibody. As shown in Fig. 6b, a specific AMA1 band appeared at ~ 66 kDa and a specific EtMIC2 band appeared at ~ 56 kDa, with no bands being detected in the vector controls. The presence of synthesized antigens on the surface of Lp-12 strains were confirmed by immunofluence assay, where green fluorescence could be observed (Fig. 6c). These results suggested that the AMA1 and EtMIC2 proteins were co-anchored on the surface of recombinant Lp-12 strains.

### Induced protection for chickens against *E. tenella*

To determine whether Lp-12-elicited immune response could provide protection against *E. tenella*, birds in experimental groups were challenged with *E. tenella* (5 × 10^4^ oocysts/bird) after booster vaccination with Lp-12 strain. Ten days after the challenge, the body weight and oocyst output of chickens were observed and lesion scores in cecum were recorded. As shown in Table 2, the chickens immunized with Lp-12 had significantly increased body weight gains (BWG), decreased cecum lesions and reduced numbers of oocyst output compared with PBS and Lp/pLQa-pgsA′ group (P < 0.001). No chickens died after challenge in any group.

**Table 2:** Protective effects in each group

Groups	Survival rate (%)	Body weight gain (g)	Relative body weight gain rate (%)	Lesion scores in cecum	Oocyst output (× 10^5^)	Oocyst decrease ratio (%)
PBS	100	337.40 ± 11.71^A^	100	0	0	100
PBS-challenge	100	210.50 ± 16.16^B^	62.39	3.83 ± 0.41^A^	9.50 ± 3.03^A^	0
Lp/pLQa-pgsA′	100	234.20 ± 22.48^C^	69.41	3.33 ± 0.82^A^	7.41 ± 2.16^A^	22.00
Lp-12	100	313.71 ± 6.60^D^	92.98	2.00 ± 0.63^B^	3.56 ± 1.30^B^	62.53

Different character means different statistical significance. Statistically significant among numbers with different characters (P > 0.05). No statistically significant among numbers with the same characters (P < 0.05). PBS group and PBS-challenge control group were treated with 200 μL PBS, p.o. The experimental (Lp-12) group was treated with Lp-12 (2 × 10^9^ CFU/200 μL), p.o. Animals in the vector control group were treated with Lp/pLQa-pgsA′ (2 × 10^9^ CFU/200 μL) at 4–6 days of age. A booster vaccination of Lp/pLQa-pgsA′ (2 × 10^9^ CFU/200 μL) was administered when animals were 18–20 days of age. Ten days after the immunization (30 days of age), chickens (other than those in the PBS control group) were challenged with 5 × 10^4^*E. tenella* sporulated oocysts

### Lp-12 improved the T cell responses in chickens

**Fig. 7:**
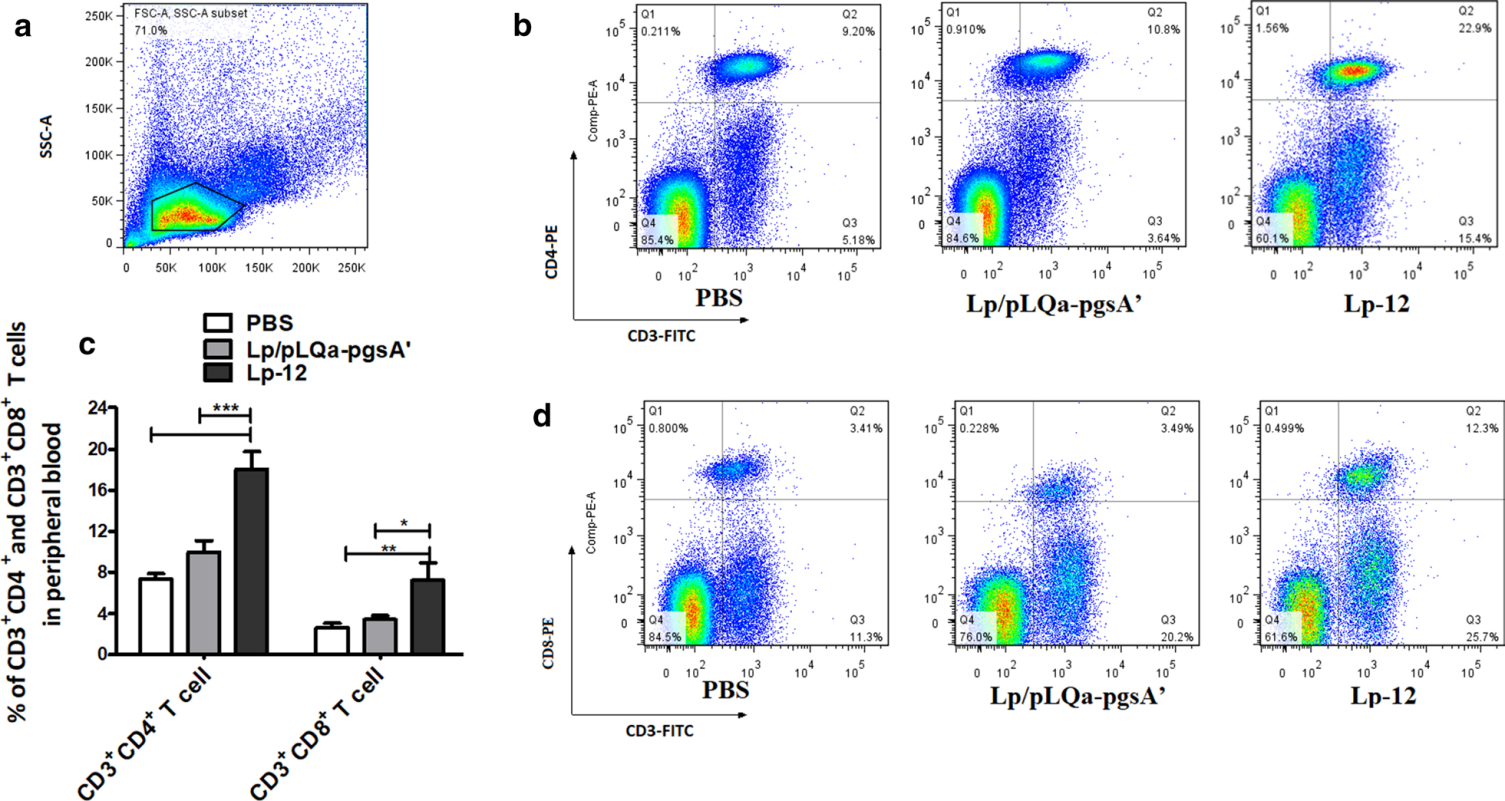
Flow cytometry analysis of Lp-12-triggered T cell responses after vaccination. **a** The single cells in peripheral-blood were prepared as described and subjected to flow cytometry assay gating was done according to [57]. **b** Panels representing CD3^+^ CD4^+^ T cells for each group. **c** The percentage of CD3^+^ CD4^+^ T cells and CD3^+^ CD8^+^ T cells from peripheral-blood were detected using flow cytometry on day 10 post second immunization by Lp-12. Data were shown as mean ± S.E.M (n = 5), were compared by a one-way ANOVA (*P < 0.05, **P < 0.01, ***P < 0.001). **d** Panels representing CD3^+^ CD8^+^ T cells for each group

To determine whether immunization of Lp-12 affects the cellular immune responses, the T lymphocytes cell responses in peripheral blood of chicken were evaluated by flow cytometry (Fig. 7a) 10 days after the second vaccination. The percentage of CD3^+^CD4^+^ T cells in Lp-12 group was significantly higher than that in Lp/pLQa-pgsA′ group and PBS group (Fig. 7b, c) (P < 0.001). Meanwhile, the proportions of CD3^+^CD8^+^ T cells in the chickens immunized with Lp-12 increased significantly compared to the Lp/pLQa-pgsA′ group and PBS group (P < 0.05) (Fig. 7c, d), There was no significant difference between PBS group and vector group.

### Lp-12 strains increased specific anti-AMA1 and EtMIC2 antibody level

**Fig. 8:**
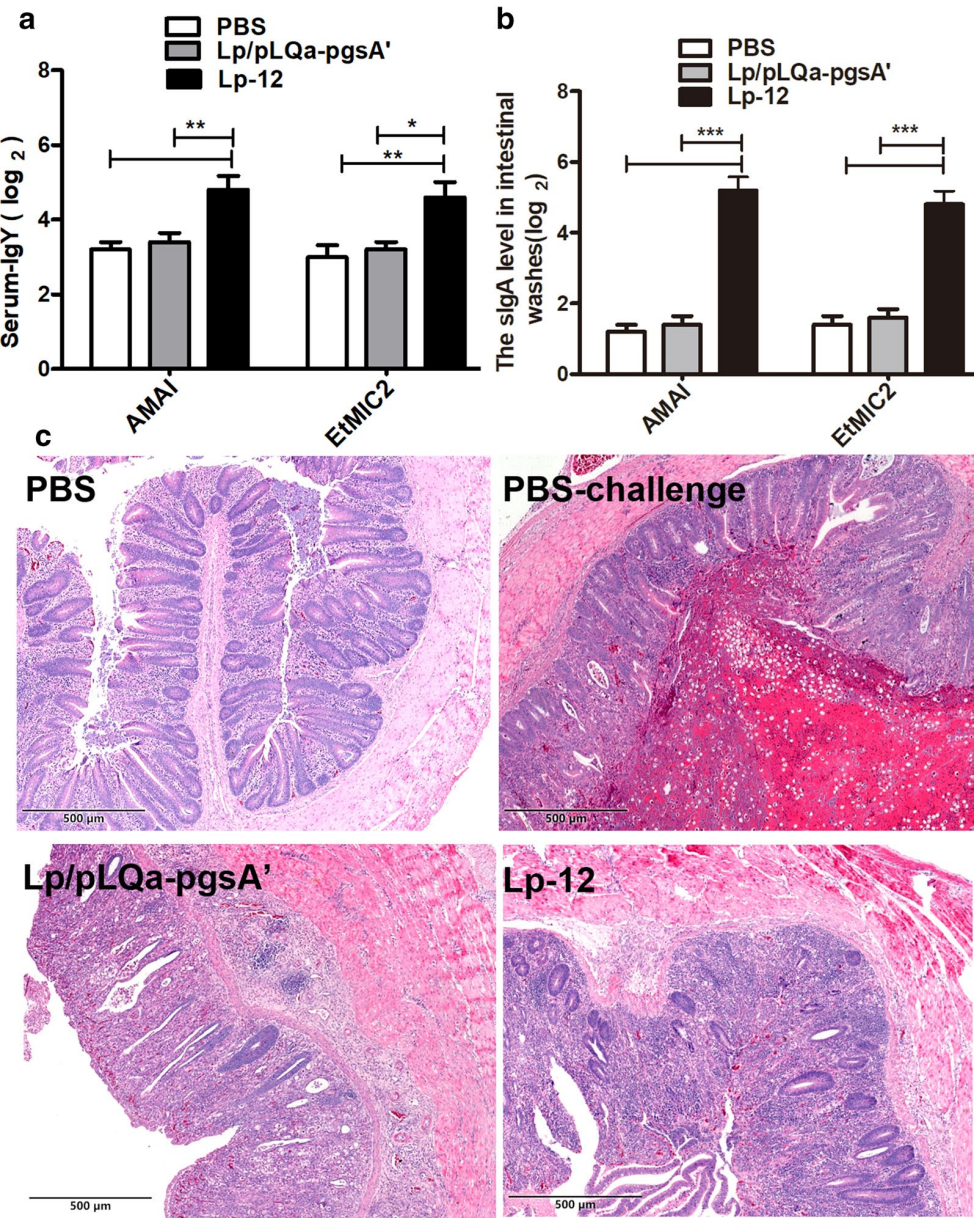
Detection of specific IgY in sera (**a**) and SIgA in intestinal washes (**b**) from chicks after boosting immunization. The data shown represent mean ± S.E.M (n = 5), which were compared by a one-way ANOVA (*P < 0.05, **P < 0.01, and ***P < 0.001). The samples were measured with 3 repeats. **c** Pathological cecum damage 7 dpi. Sample sections were stained using HE (×100 magnification); PBS: PBS control group. The villi and glands of the cecum are clearly visible and appear intact. PBS-challenge: The PBS-challenge group exhibits seriously damaged cecum villi and blurred gland was with blood cells and inflammatory cells present in the submucosa. A large number of coccidial oocysts can be found in the cecum lumen. Lp/pLQa-pgsA′: Lp/pLQa-pgsA′ immunized, followed by a challenge with *E. tenella.* The cecum villi were severely damaged and the glands appear blurred. Lp-12: Lp-12 immunized, followed by a challenge with *E. tenella*. The cecum villi were partly exfoliated, but the villus structure and the submucosal tissue remained relatively intact

To determine whether vaccination of recombination Lp-12 strains affects the specific antibody levels, 10 days after the second immunization, 5 chickens from each group were chosen to detect the specific serum IgY and intestinal SIgA antibody titers. The results showed that higher specific IgY titer against both AMA1 and EtMIC2 proteins was observed in the Lp-12 group compared with the PBS and Lp/pLQa-pgsA′ groups (Fig. 8a). Similar results were also observed regarding to the mucosal IgA antibody response (Fig. 8b), indicating that the co-expressed antigens could provide us an efficient approach for oral immunization.

### Lp-12 protected chickens from colonic inflammation

HE staining showed that chickens in the PBS-challenge group and the Lp/pLQa-pgsA′ vector group had severe colonic inflammation (Fig. 8c). The cecum villus was seriously damaged, and gland structure was blurred, with inflammatory cells and blood cells in the submucosa. The cecum had a large number of coccidial oocysts in the PBS-challenge group. The Lp-12 group showed the smallest histopathological changes. The villus of cecum was partly exfoliated, but the villus structure was relatively intact.

## Discussion

In the genetic engineering of bacteria such as *E. coli*, *Bacillus* spp. and LAB, antibiotic resistance genes have traditionally been used as selection markers for the maintenance of recombinant cells [36]. For example, numerous recombinant *L. plantarum* strains have been constructed to deliver various functional proteins such as antigens [1, 37], enzymes [38], and cytokines [39–41]. However, most of these studies used the *erm* antibiotic resistance gene as selection marker. This resistance gene may however affect human health or the environment negatively [42].

In recent years, auxotrophic selection marker genes such as *asd* [43] and *alr* [44] have been used successfully in prokaryotic expression plasmids. In this study, using an *asd*-*alr* fusion gene as an auxotrophic selection marker, we designed a series of food-grade plasmids by replacing the *erm* gene of the pLp_1261Inv-derived expression plasmids. This method can be applied to other pSIP-type vectors, which can then be used as templates for the amplification of DNA fragments when replacing the *erm* gene with the *asd*-*alr* gene. Our results indicated that the *asd*-*alr*-marked plasmids were more stable than the *erm*-marked plasmids in both selective and nonselective media. In the *alr* gene deleted host strain, the *alr* plasmid is required for growth. The non-selective medium may not have any disadvantage to the cells. In contrast, an antibiotic gene is not necessary but harmful to cells. The non-selective medium does not have selection pressure towards the host strain, so the plasmid may get lost easily. These findings are consistent with those reported previously [21]. More importantly, the *asd*-*alr*-marked plasmid allows for the screening and selection of this plasmid in *E. coli* (with *asd*) and in *L. plantarum* (with *alr*). This makes it valuable and sustainable for use in the context of LAB engineering [44].

In this study, pgsA′ and S-layer proteins were employed for the co-expression of foreign proteins in *L. plantarum*. The results showed that both EGFP and mCherry proteins were successfully co-expressed on the surface of *L. plantarum* NC8/Δalr and the fluorescence signals for EGFP and mCherry could easily be measured in bacterial suspensions. This single plasmid with two anchoring sequences was subsequently employed for the co-expression of AMA1/EtMIC2 resulting in the recombinant *L. plantarum* strain Lp-12 to exhibit an anti-coccidial effect due to the antigens delivered via the engineered strain.

To evaluate the immune effect of the Lp-12 vaccine strain, we quantified the IgY and SIgA antibody titers and percentages of T cells in peripheral blood. The anti-AMA1 or anti-EtMIC2 specific IgY and SIgA antibodies were significantly higher (P < 0.05) in Lp-12 group comparing with PBS group and vector group. The data revealed that Lp-12 can elicit strong humoral and mucosal immunity. The result is in accordance with our previous report, where a recombinant *L. plantarum* NC8 strain expressed the SO7-DCpep fusion protein by the *erm*-marked plasmid pSIP409 [1]. The proportions of CD3^+^CD4^+^ T and CD3^+^CD8^+^ T cells in peripheral blood were significantly higher in the Lp-12 group, which indicates that Lp-12 efficiently stimulates the cellular immune response, the *L. plantarum* strain with no antigen can also increase the proportion of CD3^+^CD4^+^ T and CD3^+^CD8^+^ T cells. The Lp-12 with surface-displayed AMA1/EtMIC2 might be more effective in interacting with intestinal cells to stimulate the mucosal immune response [45] than the strain with no antigen.

To further evaluate the protecting effect of the Lp-12 against coccidia, chickens were challenged with *E. tenella*. The average BWG of the group vaccinated with Lp-12 was higher compared to the PBS-challenge control group and the empty vector group (P < 0.05). Our results showed that the chickens immunized with Lp-12 exhibited reduced oocyst shedding, less cecum damage, and lower average lesion scores compared to chickens immunized with the empty vector. An explanation for our results could be that the Lp-12 vaccine was able to stimulate the cellular immune response in addition to the humoral and mucosal immune response, resulting in reduced damage of chicken gut tissue, improved feed utilization and an increase in weight gain, the protective effects obtained in this study are similar to those obtained in other studies [27, 45, 46]. One limitation of this animal study is this is a single trial. Further studies, including the protective effect with attenuated vaccines, would be helpful to further confirm these conclusions.

*Lactobacillus* has been used as a feed supplement to improve weight gain and promote health effects [47]. Previous studies have shown that *Lactobacillus* itself has an anti-coccidial effect [48]. Our current results showed the *L. plantarum* strain with no antigen (vector control group: Lp/pLQa-pgsA′) can significantly improve the body weight gain of chickens (P < 0.05) compared with the PBS-challenged group.

Attenuated vaccines against *Eimeria* parasite are mainly from egg-adapted lines or precocious strains. Because egg-adapted lines are difficult to obtain, the precocious strains are the most widely used. Although the precocious strains provide a satisfactory protective immunity, its uses are limited due to the high cost and reduction of performance [9]. In this study, we developed a safe, economic, and practical oral vaccine against *E. tenella*, using the recombinant *L. plantarum* strain as a mucosal vaccine vector. This strain offers several advantages for the development of a coccidiosis vaccine, including ease of use, low cost, higher safety levels, and feasibility of genetic manipulation [49]. To our best knowledge, this is the first report in which an engineered LAB strain delivered double-antigen induced immune responses towards *E. tenella*. Compared with attenuated vaccines, the recombinant strains may not be as effective as the attenuated vaccines in a humoral immune response, but our present results from T cells proportions suggest the function of recombinant strains might be more effective. In addition, the double-anchoring sequence based co-expression strategy may not be restricted to *E. tenella,* but provide us a novel platform to co-express antigens from other pathogens. Our technology may further expand its applications in the field of oral vaccine.

## Conclusions

In conclusion, we successfully constructed a plasmid that was used to express two foreign proteins on the surface of the *L. plantarum* NC8/Δ*alr* strain. This plasmid has two anchoring motifs for the two proteins and carries an *asd*-*alr* fusion gene as the complementation screening marker. With the non-antibiotic markers, both *E. coli* and *L. plantarum* could be screened for successful transformation events and therefore safe for human use. The recombinant Lp-12 strain with co-expressed the AMA1/EtMIC2 improved the chicken immune response and provided partial protection against *E. tenella* challenge. This may provide a novel option for parasite vaccine research.

## Methods

### Plasmids, bacterial strains and growth conditions

The plasmids and bacterial strains used are listed in Table 3. *E. coli* Top10 was grown in lysogeny broth (LB). *L. plantarum* NC8 was cultured in De Man, Rogosa and Sharpe (MRS) medium at 30 °C without agitation. *L. plantarum* NC8-competent cells were prepared as previously described [50]. The *L. plantarum* NC8/Δ*alr* strain was cultured in MRS medium with 200 μg/mL d-ala (Sigma, USA). *E. coli* χ6212 was cultured in LB medium with 50 μg/mL DAP (Sigma, USA) to compensate for the *asd* deletion. *E. coli* χ6212-competent cells were prepared and transformed as described previously [51]. Unless otherwise stated, the erythromycin concentration was 5 μg/mL (for *L. plantarum* NC8) or 200 μg/mL (for *E. coli* Top10). The AMA1 and EtMIC2 proteins were purified from *E. coli* BL21 (DE3) using a His-tagged protein purification kit (Cwbiotech, China). The mouse anti-AMA1 polyclonal antibody was obtained by immunizing mice with the purified AMA1 protein. A highly pathogenic *E. tenella* strain was isolated from chickens suffering from clinical coccisiosis at the Animal Hospital of Jilin Agricultural University and stored in our lab. In order to ensure its vitality, the *E. tenella* strain was rejuvenated in chickens every 6 months. The sporulated oocysts were preserved in 2.5% potassium dichromate solution at 4 °C as previously described [52].

**Table 3:** Strains and plasmids used in this study

Strains or plasmids	Relevant characteristics	Refs.
Strains		
*L. plantarum*		
NC8	Host strain, plasmid-free, silage isolate	[55]
NC8/Δ*alr*	d-Alanine auxotroph	This work
*E. coli*		
χ6212	*asd* auxotroph, cloning host	[51]
TOP10	Cloning host	Takara (Dalian, China)
Plasmids		
pNZ5319	Cm^r^,erm^r^, containing lox66-P32-cat-lox71 fragment	[53]
pNZ5348	erm^r^,Cre-recombinase expression	[53]
pLp_1261Inv	erm^r^; pLp_2588sAmyA derivative where the Lp_2588-AmyA cassette has been replaced by the lp_1261 and Inv fusion gene fragment	[54]
pLp-S	erm^r^, pLp_1261Inv derivative, where lp_1261 and Inv fusion gene has been replaced by the SP-linker-S_anchoring sequence of *Slp*A gene containing MCS1	This work
pLp-pgsA′	erm^r^, pLp_1261Inv derivative, where lp_1261 inv gene has been replaced by pgsA′ gene containing MCS2.	This work
pLP-pgsA′-EGFP	erm^r^, pLP-pgsA′, derivative, EGFP was cloned in XbaI/Hind III sites, containing a truncated pgsA gene, pgsA′ (567 bp)	This work
pLp-EGFP-S	erm^r^, pLp-S derivative, EGFP was cloned into SalI/XhoI sites	This work
pYA3342	Containing *asd* fragment	[57]
pLQa-pgsA′	pLP-pgsA′ derivative, the *erm* was replaced by the *asd*-*alr*	This work
pLQa-pgsA′-EGFP	pLp-pgsA′-EGFP derivative, the *erm* was replaced by the *asd*-*alr*	This work
pLQa-S	pLp-S derivative, the *erm* was replaced by the *asd*-*alr*	This work
pLQa-EGFP-S	pLp-EGFP-S derivative, the *erm* was replaced by the *asd*-*alr*	This work
pLQa-p′mCherry	pLQa-pgsA′ derivative, mCherry inserted into XbaI/HindIII-digested sites	This work
pLQa-p′m-ES	pLQa-p′mCherry derivative, SP-linker-EGFP-S_anchoring fusion gene inserted into HindIII-digested sites with introduction of the SD sequence	This work
pLQa-ES-p′m	pLQa-EGFP-S derivative, pgsA′-mCherry-His tag fusion gene inserted into HindIII sites with introduction of the SD sequence	This work
pLQa-AMA1S-p′EtMIC2	pLQa-ES-P′m derivative, AMA1 gene inserted into SalI/XhoI sites, EtMIC2 gene inserted into XbaI/HindIII-digested sites	This work

Cm^r^,erm^r^, chloramphenicol and erythromycin resistance; cre, cre-recombinase encoding gene; *asd*, aspartate β-semialdehyde dehydrogenase-encoding gene; *alr*, alanine racemase encoding gene; *erm*, erythromycin resistance gene

### DNA manipulation

The PurePlasmid Mini Kit (Cwbiotech) was used to extract plasmids, and the Gel Extraction Kit (Cwbiotech) was used to purify DNA fragments. Restriction enzymes and PrimeSTAR Max DNA Polymerase were purchased from TaKaRa (Dalian, China). Polymerase chain reaction fragments were cloned into digested plasmids using the In-Fusion HD cloning kit (Clontech Laboratories, Inc.), according to the manufacturer’s instructions. The PCR primers used are listed in Table 4. These PCR fragments were sequenced by Genewiz (Suzhou, China). Transformants harboring *asd*-*alr*^+^ plasmids were selected on LB (without DAP) or MRS (without d-ala).

**Table 4:** List of PCR primers used in the study

Primer	Primer sequences (5′–3′) for PCR sequence
P-EGFP F	GAAAATTAGTTACCAGAAAGTG*TCTAGA*ATGGTGAGCAAGGG (XbaI)
P-EGFP R	AGCAACACGTGCTGTAATTTG*AAGCTT*TTACTTGTACAGCTCGTC (HindIII)
S-EGFP F	GGCACGATTGCGGCG*GTCGAC*ATGGTGAGCAAGGGCGAGG (SalI)
S-EGFP R	GCATGGTACCGAATTC*CTCGAG*CTTGTACAGCTCGTCCATG (XhoI)
asd F	GCACCGCTATGCGTGCG*GGATCC*TCTTCCCTAAATTTAAAT (BamHI)
asd R	GTCCACAACATCAGGTAGTG
P23 F	TGTCACTACCTGATGTTGTGGACGAAAAGCCCTGACAAC
alr R	CAAATTTAAAAAAGCGACTCATAGAATTAATCTATATAAACTCTCG
pF	TTCTATGAGTCGCTTTTTTAAATTTG
pR	GGATCCCGCACGCATAGCGGTGC
SD-P′ MF	GCAAACTTTAGATAATAA*AAaCTT***AGGAAACAGACC**ATGGGCAAG AAAGAAT
SD-P′ M R	GCAACACGTGCTGTAATTTG*AAGCTT*TTAATGGTGATGGTGATGATG
SD-EGFPS F	TCATCACCATCACCATTAA*AAGCTT***AGGAAACAGACC**ATGAAGAA AAATTTAAG
SD-EGFPS R	CACGTGCTGTAATTTG*AAGCTT*TTATTATCTAAAG TTTGC

^a^Letters in italics indicate the introduction of restriction sites

^b^Underlined text indicates extensions of about 15–25 bp that were complementary to the ends of the digested vector or anchoring gene fragments for seamless cloning or overlap extension (SOE)-PCR

^c^Boldface letters indicate SD

### Construction of alr-deletion mutants

The *alr*-deletion mutant of *L. plantarum* NC8 (*L. plantarum* NC8/Δ*alr*) was constructed using the Cre-lox-based vector mutagenesis system [53] (pNZ5319/pNZ5348) as previously described [21].

### Construction of pLp-S, pLp-pgsA′, pLp-pgsA′-EGFP and pLp-EGFP-S cell-surface display vectors

Plasmids were constructed as shown in Fig. 1. First, to obtain the single-anchoring vector, the pLp-S and pLp-pgsA′ plasmids were constructed by replacing the lp_1261 Inv fusion gene of the plasmid pLp_1261Inv (Table 1) either with a part of the *Slp*A gene (SP-linker and S_anchoring sequence Genbank NO. X71412) or shortened pgsA gene (pgsA′) [35], respectively. Both genes were codon optimized and synthesized by Genewiz (Suzhou, China). A synthesized fragment containing a linker (GGCACGATTGCGGCG) [54] and the multiple cloning site MCS1 (SalI, XhoI, EcoRI, KpnI) between the SP and S-anchoring sequence was digested with NdeI/HindIII and ligated into the NdeI/HindIII digested pLp-1261Inv (5,6 kba fragment) to yield pLp-S. A synthesized fragment containing a linker (GGCACGATTGCGGCG) [54] and the multiple cloning site MCS2 (XbaI, SmaI, AsuII, NarI, and HindIII) downstream of ogsA′ was digested with NdeI/HindIII and ligated into the NdeI/HindIII digested pLp-1261Inv (5,6 kba fragment) to yield pLp-pgsA′.

EGFP (GenBank: MK317917.1) fragments were synthesized by Genewiz and amplified using P-EGFP F/P-EGFP R and S-EGFP F/S-EGFP R primers (Table 4), respectively. The PCR products were then cloned into the XbaI/HindIII-digested pLP-pgsA′ plasmid or the SalI/XhoI-digested pLp-S plasmid, respectively, yielding pLp-pgsA′-EGFP and pLp-EGFP-S plasmids. These plasmids were amplified in *E. coli* TOP10-competent cells and transformed into *L. plantarum* NC8 [55] by electroporation.

### Construction of food-grade expression vectors pLQa-pgsA′, pLQa-pgsA′-EGFP, pLQa-S, and pLQa-EGFP-S plasmids

To construct food-grade expression vectors, the *erm* marker was replaced with the non-antibiotic marker *asd*-*alr* in the “pLp” vectors. The pYA3342 plasmid (Table 3) was used as template to amplify the *asd* gene (containing the promoter and terminator sequences) with *asd* F/*asd* R primer pairs (Table 4). A plasmid containing the *Streptococcus cremoris* promoter 23 sequence (P23, GenBank: M24763.1) and the *alr* gene (NCBI: NC_004567.2) was synthesized by Genewiz. The SalI and HindIII sites in the *alr* gene were removed during synthesis by mutation (GTCGAC → GTgGAC and AAGCTT → AAaCTT). The synthesized fragment was used as a template to amplify the P23-*alr* fusion fragment using P23 F/alr R primers (Table 4). Purified *asd* and P23-*alr* fragments were fused by overlap extension-PCR using *asd* F/*alr* R primers. Four plasmids constructed as described above (pLP-pgsA′, pLP-pgsA′-EGFP, pLp-S, and pLp-EGFP-S) were amplified without the *erm* gene using pF/pR primers (Table 4). These fragments were subsequently ligated with the *asd*-p23-*alr* fusion fragments by seamless cloning, yielding pLQa-pgsA′, pLQa-pgsA′-EGFP, pLQa-S, and pLQa-EGFP-S plasmids (Table 3, Fig. 1). These expression vectors were amplified in *E. coli* χ6212-competent cells using the *asd* gene as auxotrophic marker before transformation into the *L. plantarum* NC8/Δ*alr* strain (using *alr* gene as screening marker) by electroporation.

### Analysis of EGFP expression

To examine whether the non-antibiotic marker affects bacterial growth, six strains (*L. plantarum* NC8 harboring pLP-pgsA′, pLP-pgsA′-EGFP, and pLp-EGFP-S; *L. plantarum* NC8/Δ*alr* harboring pLQa-pgsA′, pLQa-pgsA′-EGFP, and pLQa-EGFP-S) were grown at 30 °C without agitation. The overnight liquid cultures were sub-cultured in fresh MRS to a density corresponding to an OD_600_ ≈ 0.3. Each culture was then divided into an induced culture (addition of SppIp, 25 ng/mL) and a non-induced culture. OD_600_ values were measured 4 h post induction.

To compare the expression levels of EGFP in antibiotic and non-antibiotic resistance conveying plasmids, after induction for 4 h, the density of cultures were normalized to the same OD_600_ values. Cells were harvested from the cultures, and then the protein extractions were performed by a method described previously [33, 35]. The protein in the cell-free extracts was quantified using a BCA kit (Beyotime, Shanghai, China) and same amount of protein was subjected to 12% SDS-PAGE and western blotting using mouse anti-EGFP monoclonal antibody (1:5000 dilution) (BBI Life Science) and goat anti-mouse HRP-conjugated monoclonal antibody (1:8000 dilution) (BBI Life Science). The specific band representing EGFP was quantified using ImageJ software. An inverted fluorescence microscope (Leica DMI8) was used to visualize EGFP. The EGFP was quantified using a flow cytometer (BD LSRFortessa). Strains with the empty vector pLp-pgsA′ and pLQa-pgsA′ were used as negative controls.

### Measurement of plasmid stability

Replica plating was used to detect the *plasmid stability of* pLp-pgsA′-EGFP, pLp-EGFP-S, pLQa-pgsA′-EGFP, and pLQa-EGFP-S. The percentage of host cells still containing the plasmid was measured after 50 and 100 generations [21].

### Construction of a single plasmid co-expression EGFP and mCherry using dual-anchoring sequences

The mCherry gene (GenBank: KP238582.1) with 6 × His tag was synthesized by Genewiz, and was then ligated with the XbaI/HindIII-digested pLQa-pgsA′ using T_4_ DNA ligase (NEB). This process yielded the pLQa-pgsA′-mCherry plasmid (Fig. 4a). Then the SP-linker-EGFP-S_anchoring and pgsA′-mCherry fragments were amplified from pLQa-EGFP-S and pLQa-pgsA′-mCherry using SD-EGFPSF/SD-EGFPS R and SD-P′M F/SD-P′ M R primers, respectively. The amplified fragments were cloned into HindIII-digested pLQa-pgsA′-mCherry and pLQa-EGFP-S plasmids (Table 3) with introduction of the SD sequences (AGGAAACAGACC) by seamless cloning. This process yielded the two single co-expression plasmids pLQa-p′m-ES and pLQa-ES-p′m, respectively (Fig. 4a). In order to simplify further plasmid manipulations, the HindIII sites upstream the SD sequence were mutated (AAGCTT → AAaCTT) using the primer SD-P′M F (Table 4) when amplifying the pgsA′-mCherry gene. These plasmids were amplified in *E. coli* χ6212-competent cells, then transformed into *L. plantarum* NC8/Δ*alr* by electroporation.

### Co-anchoring of EGFP and mCherry on *L. plantarum* NC8/Δalr surface

*Lactobacillus plantarum* NC8/Δalr strains harboring pLQa-p′m-ES, or pLQa-ES-p′m were cultured and induced in MRS, fluorescence microscopy and flow cytometry were performed to measure EGFP and mCherry expression as described above. For Western blot analysis, 2 mL of bacterial cultures were harvested. Two identical 12% SDS-PAGE gels were run in parallel, then transferred to two nitrocellulose membranes. One was used to detect EGFP as described above. The second membrane was incubated in mouse anti-6 × His tag monoclonal antibody (1:2000 dilution) (Kangwei Co., China) and goat anti-mouse HRP-conjugated monoclonal antibody (1:8000 dilution) (BBI Life Science). Levels of protein expression were analyzed and quantified using ImageJ software. Single-expression vectors (pLQa-EGFP-S and pLQa-pgsA′-mCherry) were used as positive controls.

### Co-anchoring of AMA1and EtMIC2 on *L. plantarum* NC8/Δ*alr* surface

Both the AMA1 (GenBank: AEJ33058.1) and EtMIC2 (GenBank: ACN93990.1) fragments without signal sequence were codon optimized, synthesized by Genewiz (Suzhou, China) and cloned into the pUC57-Kan plasmid. To co-anchor AMA1 and EtMIC2, the synthesized fragments were inserted into pLQa-ES-p′m in two steps. First, AMA1 was cloned into pLQa-ES-p′m using the SalI/XhoI restriction sites, yielding pLQa-AMA1S-p′m. Second, the EtMIC2 fragments were cloned into pLQa-AMA1S-p′m using XbaI/HindIII. This process yielded pLQa-AMA1S-p′ EtMIC2 (pLQa12) (Fig. 6a). The *L. plantarum* NC8/Δalr containing the pLQa12 is referred to as Lp-12. Western blotting was used to determinate the co-expression of AMA1 and EtMIC2 using a mouse anti-AMA1 polyclonal antibody (1:2000 dilution, provided by Jilin Agricultural University) and a mouse anti-EtMIC2 monoclonal antibody (1:2000 dilution, provided by Professor Xiaomin Zhao of Shandong Agricultural University). Co-anchored AMA1 and EtMIC2 on the surface of Lp-12 was detected by an immunofluorescence assay [56]. Approximately 10^6^ cells were incubated with the mouse anti-EtMIC2 monoclonal or the anti-AMA1 polyclonal antibody, followed by incubation with FITC-conjugated goat anti-mouse IgG. Cells were washed twice with PBST and visualized using a Leica DMI8 fluorescence microscope.

### Animal experiments

All animal experiments were approved by the Animal Care and Ethics Committees of Jilin Agriculture University. White feather broiler chickens (1-day-old) were obtained from Hongda Animal Technology Co., Ltd. (Changchun, China). Chickens were weighed and randomly divided into groups A-D with 20 animals per group. All animals in the experimental group A were gavaged with 200 µL buffer containing 2 × 10^9^ CFU of Lp-12 at an age of 4 to 6 days, followed by a booster vaccination at an age of 18 to 20 days with the same amount of Lp-12. Animals in the experimental group B (vector control group) were gavaged with 200 µL buffer containing 2 × 10^9^ CPU of Lp/pLQa-pgsA′ at an age of 4 to 6 days, followed by a booster vaccination at age of 18 to 20 days with the same amount. Animals in the experimental group C (PBS control group) were gavaged with 200 µL PBS at an age of 4 to 6 days and 18 to 20 days. Animals in the experimental group D (PBS-challenge group) were treated with the same amount of PBS as group C. Ten days after the immunization (at age of 30 days), 5 chickens each of group A, B and C were chosen for sample collections to detect immune response. The remaining chickens within groups A, B and D were challenged with 5 × 10^4^ sporulated oocysts of *E. tenella* as previously described [1].

### Detection of antibody response

ELISA was performed to determine the IgY responses against AMA1 and EtMIC2 in sera, as well as secretory immunoglobulin (SIgA) in intestinal washes as described previously [14]. Briefly, to get the intestinal washes [22], the intestines were opened, and the contents were removed. Intestinal scrapings were collected using glass slides and diluted in cold PBS. After shaking for 1 min, the samples were centrifuged for 5 min at 5000×*g* at 4 °C. Supernatants were collected and stored at − 80 °C for SIgA assays. The 96-well plates were coated with AMA1 or EtMIC2 (100 ng/well) at 4 °C overnight. 1% BSA was added to each sample well and incubated overnight at 4 °C. 100 μL serum samples (1:100) and intestinal washes (1:20) were added to each well and incubated for 1.5 h at 37 °C. Subsequently, HRP-conjugated goat anti-chicken IgY or IgA (SouthernBiotech, USA) antibodies (1:10,000) were added to each well and incubated for additionally 1 h. After washing, 100 μL of 3,3′,5,5′tetramethylbenzidine (TMB) was added to each well. The reaction was stopped by addition of 2 N H_2_SO_4_ (50 μL/well). Finally, the absorbance was recorded at 450 nm in a plate reader.

### Flow cytometry

Single-cell suspensions from blood were prepared using a peripheral-blood lymphocyte separation kit (Solarbio, Beijing, China), and flow cytometry was performed to detect the percentages of CD3^+^CD4^+^, CD3^+^CD8^+^ T-cells [57]. The isolated cells (1 × 10^6^) were incubated with FITC-conjugated anti-chicken CD3 and PE-conjugated anti-chicken CD4 or CD8 (SouthernBiotech) monoclonal antibodies, respectively. The samples were quantified by a flow cytometer (BD LSRFortessa™, USA). Experiments were performed in triplicates and the data were analyzed using FlowJo (Version 7.6.2, Becton, Dickinson and Company, USA).

### Evaluation of immune protection

At 7 days post-infection (7 dpi), the cecal lesion scores of five chickens per experimental group were assessed as previously described [58]. Ceca were removed and fixed with 4% paraformaldehyde and stained with standard hematoxylin and eosin (HE) as described previously [59]. Body weights were measured at the day of challenge and 7 dpi to calculate the relative BWG (%) [9]. Fecal samples were collected between 4 dpi and 7 dpi to determine the numbers of oocysts per gram (OPG) in feces [1] and to determine the percentual reduction of oocysts in the feces of the respective treatments relative to the control [60].

### Statistical analysis

All results are presented as mean ± standard error of the mean (SEM). Data were analyzed with GraphPad Prism 5.0 software using One-Way ANOVA. P < 0.05 was considered statistically significant.

## Abbreviations

EGFP: Enhanced green fluorescent protein
*L. plantarum*: *Lactobacillus plantarum*
*E. coli*: *Escherichia coli*
*E. tenella*: *Eimeria tenella*
LAB: Lactic acid bacteria
pgsA: Poly-γ-glutamic acid synthetase A
SP: Signal peptide
S: Anchoring sequence of the surface layer protein
DAP: 5 2, 6-Diaminopimelic acid
*erm*: Erythromycin resistance gene
*alr*: d-Alanine racemase gene
*asd*: β-Semialdehyde dehydrogenase gene
d-ala: d-Alanine
EtMIC2: *Eimeria tenella* microneme-2
AMA1: Apical membrane antigen 1
SD: Shine-Dalgarno
LB: Luria broth
MRS: De Man Rogosa Sharpe
SDS: Sodium dodecyl sulfate
SDS-PAGE: SDS-polyacrylamide gel electrophoresis
PBS: Phosphate buffered saline
TMB: Tetramethylbenzidine
ELISAs: Enzyme-linked immunosorbent assay
SIgA: Secretory immunoglobulin A

## References

[1] Yang G, Yao J, Yang W, Jiang Y, Du J, Huang H, Gu W, Hu J, Ye L, Shi C (2017). Construction and immunological evaluation of recombinant *Lactobacillus plantarum* expressing SO7 of *Eimeria tenella* fusion DC-targeting peptide. Vet Parasitol.

[2] Liu L, Zhang W, Song Y, Wang W, Zhang Y, Wang T, Li K, Pan Q, Qi X, Gao Y (2018). Recombinant *Lactococcus lactis* co-expressing OmpH of an M cell-targeting ligand and IBDV-VP2 protein provide immunological protection in chickens. Vaccine.

[3] Shirley MW, Bedrnik P (1997). Live attenuated vaccines against avian coccidiosis: success with precocious and egg-adapted lines of Eimeria. Parasitol Today.

[4] Li GQ, Kanu S, Xiao SM, Xiang FY (2005). Responses of chickens vaccinated with a live attenuated multi-valent ionophore-tolerant Eimeria vaccine. Vet Parasitol.

[5] Yin G, Lin Q, Wei W, Qin M, Liu X, Suo X, Huang Z (2014). Protective immunity against *Eimeria tenella* infection in chickens induced by immunization with a recombinant C-terminal derivative of EtIMP1. Vet Immunol Immunopathol.

[6] Lin Z, Shi Y, Deng B, Mao X, Yu D, Li W (2015). Protective immunity against *Eimeria tenella* infection in chickens following oral immunization with *Bacillus subtilis* expressing *Eimeria tenella* 3-1E protein. Parasitol Res.

[7] Li WC, Zhang XK, Du L, Pan L, Gong PT, Li JH, Yang J, Li H, Zhang XC (2013). *Eimeria maxima*: efficacy of recombinant *Mycobacterium bovis* BCG expressing apical membrane antigen1 against homologous infection. Parasitol Res.

[8] Shivaramaiah S, Barta JR, Layton SL, Lester C, Tellez G (2010). Development and evaluation of an Δ aroA/Δ htrA *Salmonella enteritidis* vector expressing *Eimeria maxima* TRAP family protein EmTFP250 with CD 154 (CD 40L) as candidate vaccines against coccidiosis in broilers. Int J Poult Sci..

[9] Ma D, Gao M, Dalloul RA, Ge J, Ma C, Li J (2013). Protective effects of oral immunization with live *Lactococcus lactis* expressing *Eimeria tenella* 3-1E protein. Parasitol Res.

[10] Lu W, Kong J, Kong W (2013). Construction and application of a food-grade expression system for *Lactococcus lactis*. Mol Biotechnol.

[11] Yoon SW, Lee TY, Kim SJ, Lee IH, Sung MH, Park JS, Poo H (2012). Oral administration of HPV-16 L2 displayed on *Lactobacillus casei* induces systematic and mucosal cross-neutralizing effects in Balb/c mice. Vaccine.

[12] Lei H, Peng X, Zhao D, Ouyang J, Jiao H, Shu H, Ge X (2015). *Lactococcus lactis* displayed neuraminidase confers cross protective immunity against influenza A viruses in mice. Virology.

[13] Smit E, Jager D, Martinez B, Tielen FJ, Pouwels PH (2002). Structural and functional analysis of the S-layer protein crystallisation domain of *Lactobacillus acidophilus* ATCC 4356: evidence for protein-protein interaction of two subdomains. J Mol Biol.

[14] Jiang Y, Yang G, Wang Q, Wang Z, Yang W, Gu W, Shi C, Wang J, Huang H, Wang C (2017). Molecular mechanisms underlying protection against H9N2 influenza virus challenge in mice by recombinant *Lactobacillus plantarum* with surface displayed HA2-LTB. J Biotechnol.

[15] Yang WT, Li QY, Ata EB, Jiang YL, Huang HB, Shi CW, Wang JZ, Wang G, Kang YH, Liu J (2018). Immune response characterization of mice immunized with *Lactobacillus plantarum* expressing spike antigen of transmissible gastroenteritis virus. Appl Microbiol Biotechnol.

[16] Jin YB, Yang WT, Shi CW, Feng B, Huang KY, Zhao GX, Li QY, Xie J, Huang HB, Jiang YL (2018). Immune responses induced by recombinant *Lactobacillus plantarum* expressing the spike protein derived from transmissible gastroenteritis virus in piglets. Appl Microbiol Biotechnol.

[17] Huang KY, Yang GL, Jin YB, Liu J, Chen HL, Wang PB, Jiang YL, Shi CW, Huang HB, Wang JZ (2018). Construction and immunogenicity analysis of *Lactobacillus plantarum* expressing a porcine epidemic diarrhea virus S gene fused to a DC-targeting peptide. Virus Res.

[18] Trombert A (2015). Recombinant lactic acid bacteria as delivery vectors of heterologous antigens: the future of vaccination?. Benef Microbes.

[19] Zhu D, Zhao K, Xu H, Zhang X, Bai Y, Saris PEJ, Qiao M (2014). Construction of thyA deficient *Lactococcus lactis* using the Cre-loxP recombination system. Ann Microbiol.

[20] Glenting J, Madsen SM, Vrang A, Fomsgaard A, Israelsen H (2002). A plasmid selection system in *Lactococcus lactis* and its use for gene expression in *L. lactis* and human kidney fibroblasts. Appl Environ Microbiol.

[21] Nguyen TT, Mathiesen G, Fredriksen L, Kittl R, Nguyen TH, Eijsink VG, Haltrich D, Peterbauer CK (2011). A food-grade system for inducible gene expression in *Lactobacillus plantarum* using an alanine racemase-encoding selection marker. J Agric Food Chem.

[22] Jiang Y, Mo H, Willingham C, Wang S, Park JY, Kong W, Roland KL, Curtiss R (2015). Protection against necrotic enteritis in broiler chickens by regulated delayed lysis Salmonella vaccines. Avian Dis.

[23] Zhang TE, Yin LT, Li RH, Wang HL, Meng XL, Yin GR (2015). Protective immunity induced by peptides of AMA1, RON2 and RON4 containing T-and B-cell epitopes via an intranasal route against toxoplasmosis in mice. Parasite Vectors.

[24] Pastor-Fernandez I, Kim S, Billington K, Bumstead J, Marugan-Hernandez V, Kuster T, Ferguson DJP, Vervelde L, Blake DP, Tomley FM (2018). Development of cross-protective Eimeria-vectored vaccines based on apical membrane antigens. Int J Parasitol.

[25] Jiang L, Lin J, Han H, Dong H, Zhao Q, Zhu S, Huang B (2012). Identification and characterization of *Eimeria tenella* apical membrane antigen-1 (AMA1). PLoS ONE.

[26] Hoan TD, Thao DT, Gadahi JA, Song X, Xu L, Yan R, Li X (2014). Analysis of humoral immune response and cytokines in chickens vaccinated with *Eimeria brunetti* apical membrane antigen-1 (EbAMA1) DNA vaccine. Exp Parasitol.

[27] Li J, Wang F, Ma C, Huang Y, Wang D, Ma D (2018). Recombinant *Lactococcus lactis* expressing *Eimeria tenella* AMA1 protein and its immunological effects against homologous challenge. Exp Parasitol.

[28] Sun H, Wang L, Wang T, Zhang J, Liu Q, Chen P, Chen Z, Wang F, Li H, Xiao Y, Zhao X (2014). Display of *Eimeria tenella* EtMic2 protein on the surface of *Saccharomyces cerevisiae* as a potential oral vaccine against chicken coccidiosis. Vaccine.

[29] Shi W, Liu Q, Zhang J, Sun J, Jiang X, Geng J, Wang F, Xiao Y, Li H, Zhao X (2014). Co-expression of EtMic2 protein and chicken interleukin-18 for DNA vaccine against chicken coccidiosis. Res Vet Sci.

[30] Ding X, Lillehoj HS, Dalloul RA, Min W, Sato T, Yasuda A, Lillehoj EP (2005). In ovo vaccination with the *Eimeria tenella* EtMIC2 gene induces protective immunity against coccidiosis. Vaccine.

[31] Yan M, Cui X, Zhao Q, Zhu S, Huang B, Wang L, Zhao H, Liu G, Li Z, Han H, Dong H (2018). Molecular characterization and protective efficacy of the microneme 2 protein from *Eimeria tenella*. Parasite.

[32] Kuczkowska K, Mathiesen G, Eijsink VG, Oynebraten I (2015). *Lactobacillus plantarum* displaying CCL3 chemokine in fusion with HIV-1 Gag derived antigen causes increased recruitment of T cells. Microb Cell Fact.

[33] Dieye Y, Usai S, Clier F, Gruss A, Piard JC (2001). Design of a protein-targeting system for lactic acid bacteria. J Bacteriol.

[34] Lin J, Zou Y, Ma C, Liang Y, Ge X, Chen Z, She Q (2016). Construction and characterization of three protein-targeting expression system in *Lactobacillus casei*. FEMS Microbiol Lett.

[35] Cai R, Jiang Y, Yang W, Shi S, Shi C, Hu J, Gu W, Ye L, Zhou F, Gong Q (2016). Surface-displayed IL-10 by recombinant *Lactobacillus plantarum* reduces Th1 responses of RAW264.7 cells stimulated with poly(I:C) or LPS. J Microbiol Biotechnol.

[36] Wen LJ, Hou XL, Wang GH, Yu LY, Wei XM, Liu JK, Liu Q, Wei CH (2012). Immunization with recombinant *Lactobacillus casei* strains producing K99, K88 fimbrial protein protects mice against enterotoxigenic *Escherichia coli*. Vaccine.

[37] Shi SH, Yang WT, Yang GL, Zhang XK, Liu YY, Zhang LJ, Ye LP, Hu JT, Xing X, Qi C (2016). *Lactobacillus plantarum* vaccine vector expressing hemagglutinin provides protection against H9N2 challenge infection. Virus Res.

[38] Mathiesen G, Sveen A, Brurberg MB, Fredriksen L, Axelsson L, Eijsink VG (2009). Genome-wide analysis of signal peptide functionality in *Lactobacillus plantarum* WCFS1. BMC Genom.

[39] Yang G, Jiang Y, Yang W, Du F, Yao Y, Shi C, Wang C (2015). Effective treatment of hypertension by recombinant *Lactobacillus plantarum* expressing angiotensin converting enzyme inhibitory peptide. Microb Cell Fact.

[40] Michon C, Kuczkowska K, Langella P, Eijsink VG, Mathiesen G, Chatel JM (2015). Surface display of an anti-DEC-205 single chain Fv fragment in *Lactobacillus plantarum* increases internalization and plasmid transfer to dendritic cells in vitro and in vivo. Microb Cell Fact.

[41] Yang WT, Yang GL, Wang Q, Huang HB, Jiang YL, Shi CW, Wang JZ, Huang KY, Jin YB, Wang CF (2017). Protective efficacy of Fc targeting conserved influenza virus M2e antigen expressed by *Lactobacillus plantarum*. Antiviral Res.

[42] Renault P (2002). Genetically modified lactic acid bacteria: applications to food or health and risk assessment. Biochimie.

[43] Zhao X, Dai Q, Zhu D, Liu M, Chen S, Sun K, Yang Q, Wu Y, Kong Q, Jia R (2017). Recombinant attenuated *Salmonella Typhimurium* with heterologous expression of the *Salmonella choleraesuis O*-polysaccharide: high immunogenicity and protection. Sci Rep.

[44] Staudigl P, Haltrich D, Peterbauer CK (2014). l-Arabinose isomerase and d-xylose isomerase from *Lactobacillus reuteri*: characterization, coexpression in the food grade host *Lactobacillus plantarum*, and application in the conversion of d-galactose and d-glucose. J Agric Food Chem.

[45] Ma C, Zhang L, Gao M, Ma D (2017). Construction of *Lactococcus lactis* expressing secreted and anchored *Eimeria tenella* 3-1E protein and comparison of protective immunity against homologous challenge. Exp Parasitol.

[46] Wang Q, Chen L, Li J, Zheng J, Cai N, Gong P, Li S, Li H, Zhang X (2014). A novel recombinant BCG vaccine encoding *Eimeria tenella* rhomboid and chicken IL-2 induces protective immunity against coccidiosis. Korean J Parasitol.

[47] Trabelsi I, Ktari N, Ben Slima S, Bouchaala K, Ben Salah R (2016). Effects of supplementation with *L. plantarum* TN8 encapsulated in alginate-chitosan in broiler chickens. Int J Biol Macromol.

[48] Giannenas I, Papadopoulos E, Tsalie E, Triantafillou E, Henikl S, Teichmann K, Tontis D (2012). Assessment of dietary supplementation with probiotics on performance, intestinal morphology and microflora of chickens infected with *Eimeria tenella*. Vet Parasitol.

[49] Cano-Garrido O, Seras-Franzoso J, Garcia-Fruitos E (2015). Lactic acid bacteria: reviewing the potential of a promising delivery live vector for biomedical purposes. Microb Cell Fact.

[50] Josson K, Scheirlinck T, Michiels F, Platteeuw C, Stanssens P, Joos H, Dhaese P, Zabeau M, Mahillon J (1989). Characterization of a gram-positive broad-host-range plasmid isolated from *Lactobacillus hilgardii*. Plasmid.

[51] Galan JE, Nakayama K, Curtiss R (1990). Cloning and characterization of the asd gene of *Salmonella typhimurium*: use in stable maintenance of recombinant plasmids in Salmonella vaccine strains. Gene.

[52] Yang S-y (2017). Letting the troops loose: pillage, massacres, and enslavement in early tang warfare. J Chin Mil Hist.

[53] Lambert JM, Bongers RS, Kleerebezem M (2007). Cre-lox-based system for multiple gene deletions and selectable-marker removal in *Lactobacillus plantarum*. Appl Environ Microbiol.

[54] Fredriksen L, Kleiveland CR, Hult LT, Lea T, Nygaard CS, Eijsink VG, Mathiesen G (2012). Surface display of N-terminally anchored invasin by *Lactobacillus plantarum* activates NF-kappaB in monocytes. Appl Environ Microbiol.

[55] Sorvig E, Mathiesen G, Naterstad K, Eijsink VG, Axelsson L (2005). High-level, inducible gene expression in *Lactobacillus sakei* and *Lactobacillus plantarum* using versatile expression vectors. Microbiology.

[56] Yang WT, Yang GL, Zhao L, Jin YB, Jiang YL, Huang HB, Shi CW, Wang JZ, Wang G, Kang YH, Wang CF (2018). *Lactobacillus plantarum* displaying conserved M2e and HA2 fusion antigens induces protection against influenza virus challenge. Appl Microbiol Biotechnol.

[57] Gao X, Xu K, Yang G, Shi C, Huang H, Wang J, Yang W, Liu J, Liu Q, Kang Y (2019). Construction of a novel DNA vaccine candidate targeting F gene of genotype VII Newcastle disease virus and chicken IL-18 delivered by Salmonella. J Appl Microbiol.

[58] Johnson J, Reid WM (1970). Anticoccidial drugs: lesion scoring techniques in battery and floor-pen experiments with chickens. Exp Parasitol.

[59] Jiang Y, Hu J, Guo Y, Yang W, Ye L, Shi C, Liu Y, Yang G, Wang C (2015). Construction and immunological evaluation of recombinant *Lactobacillus plantarum* expressing HN of Newcastle disease virus and DC-targeting peptide fusion protein. J Biotechnol.

[60] Song X, Zhao X, Xu L, Yan R, Li X (2017). Immune protection duration and efficacy stability of DNA vaccine encoding *Eimeria tenella* TA4 and chicken IL-2 against coccidiosis. Res Vet Sci.

